# Recent Advances in Nanomaterial-Based Wound-Healing Therapeutics

**DOI:** 10.3390/pharmaceutics12060499

**Published:** 2020-05-30

**Authors:** Atanu Naskar, Kwang-sun Kim

**Affiliations:** Department of Chemistry and Chemistry Institute for Functional Materials, Pusan National University, Busan 46241, Korea; atanunaskar@pusan.ac.kr

**Keywords:** nanomedicine, wound healing, scaffold, nanocarriers, nanoparticles, innovative strategies, dressings infectious control

## Abstract

Nanomaterial-based wound healing has tremendous potential for treating and preventing wound infections with its multiple benefits compared with traditional treatment approaches. In this regard, the physiochemical properties of nanomaterials enable researchers to conduct extensive studies on wound-healing applications. Nonetheless, issues concerning the use of nanomaterials in accelerating the efficacy of existing medical treatments remain unresolved. The present review highlights novel approaches focusing on the recent innovative strategies for wound healing and infection controls based on nanomaterials, including nanoparticles, nanocomposites, and scaffolds, which are elucidated in detail. In addition, the efficacy of nanomaterials as carriers for therapeutic agents associated with wound-healing applications has been addressed. Finally, nanomaterial-based scaffolds and their premise for future studies have been described. We believe that the in-depth analytical review, future insights, and potential challenges described herein will provide researchers an up-to-date reference on the use of nanomedicine and its innovative approaches that can enhance wound-healing applications.

## 1. Introduction

Wound healing is the highly coordinated process of restoring damaged tissue that comprises four sequential, yet overlapping, biological stages: hemostasis, inflammation, proliferation, and remodeling [1,2,3]. Any perturbation by both external and internal factors in any of the above wound-healing phases may prolong each stage and lead to an unsatisfactory outcome, further resulting in chronic wound status. The most frequently encountered issue concerning complete wound healing is the colonization of contaminating pathogens at the site of skin injury during the natural wound-healing process [4]. The bacteria that make up the skin microbiota at the site of a wound protect against the colonization of pathogens. However, when pathogenic bacteria exceed a critical level and produce a considerable amount of biofilm, the healing process is retarded. *Staphylococcus aureus* is the most frequently identified colonizing pathogens that influence the initial phases of wound healing, whereas *Pseudomonas aeruginosa* and *Escherichia coli* are typically found in chronic wounds and affect deeper layers of the skin [5]. This pathogen-associated bare skin infection may contribute to severe inflammatory reactions and incomplete healing of wounds. Therefore, an appropriate antimicrobial-associated wound dressing must be used to prevent bacterial infections and aid in the natural wound-healing process. The explosion of antimicrobial-resistant bacteria, however, delays the healing process, thus necessitating the need to develop novel wound-dressing materials, which are non-toxic or resistant to use, and improve the efficacy of the wound-healing process.

Commercial or conventional dressings used for wound healing are usually dry in nature, typically uncomfortable, and not user friendly to use. So far, there are only four Food and Drug Administration (FDA)-approved treatment modalities for treating chronic skin wounds [6]. These include two dermal substitutes (Graftskin [7], Dermagraft [8]), a bioengineered human skin equivalent (Integra Dermal Regeneration Template, IDRT [9]), and a recombinant human platelet-derived growth factor (Becaplermin [10]). Given their impact on chronic wound healing, 44–70% of patients with chronic ulcers and treated with the above-mentioned therapies remain unhealed [11,12]. Therefore, alternative effective therapies are urgently required, which can provide precise outcomes in the wound-healing process, such as wound closure, and control of fluid loss with properties including durability, elasticity, and biocompatibility.

In the current scenario, nanomaterial-based wound-healing approaches have emerged as successful weapons against bacterial infections and provide other benefits, such as cell-type specificity, which was not previously feasible with conventional wound-dressing materials or present therapies [6,13,14]. Nanomaterials are already being applied in various biomedical applications, including biosensing, bioimaging, drug delivery, anti-cancer activity, antibacterial activity, medical diagnosis, medical equipment, food industry, cosmetics, environmental remedy, and wound healing [15,16,17,18]. The main reason for their widespread use in biomedical applications is their nanoscale (1–100 nm) and large surface area-to-volume ratio, along with suitable physicochemical properties. In particular, the nano size and high surface area-to-volume ratio allow them to communicate efficiently with the wound area and easily penetrate the skin layer at the wound site. Hence, nanomaterials not only serve as therapeutic agents to heal the wound but can also provide the wound site with a sustained and controlled release of therapeutics.

Nanomaterial-based approaches were intended to develop new alternative antibacterial agents that could kill various pathogenic bacteria. For example, metal or metal oxide nanoparticles (e.g., Ag, Au, and ZnO) were synthesized for intrinsic antibacterial activity and potential wound-healing properties [14,19]. Some nanomaterials can also direct therapeutic agents to the target site, such as liposomes [20]. In addition, some alternative approaches to detection, with the exposure of external energy sources such as near-infrared (NIR) light [21] or alternate magnetic field (AMF) [22], have been employed with Au nanoparticles, γ-Fe_2_O_3_, or Fe_3_O_4_ for irreversible thermal damage to the target cell in wound-healing applications.

In the present review, recent advances in nanomaterial-based wound-healing applications and their benefits to traditional wound-healing approaches have been highlighted. The use of nanomaterials as intrinsic antibacterial agents and as vehicles for the conveyance of therapeutic agents is discussed in sequence. In addition, the role of nanomaterials in scaffold-mediated wound healing is discussed. Finally, recent groundbreaking approaches such as hyperthermia treatment and gene nanotherapy are highlighted.

## 2. Wound Infection

### 2.1. Types of Wounds

Depending on the duration and methods of the healing process, wounds are generally classified into two categories: *acute* and *chronic wounds* [20]. Wounds resulting from corrosive chemicals, radioactivity, mechanical injury, heat, or electrical shock are considered acute wounds; they usually heal with proper wound-care treatment in a fairly short period of time. Chronic wounds, however, are associated with specific diseases, such as diabetes mellitus, and do not follow the orderly set of stages and predictable amount of time that characterize the normal wound-healing process. Chronic wounds frequently remain in the inflammatory stage for a long time, and their duration is associated with factors such as bacterial load, necrotic tissue, and moisture balance of the wound site. Further, the risks of this reappearance of chronic wounds are exceedingly high, unless the root of the disease is cured [23]. In fact, chronic wounds can never heal or may take several years to heal. Therefore, it is important to resolve the stage and to remove such inhibitory factors.

Wounds can also be classified into three types according to wound depth: (1) superficial wounds, which lost a part of epidermis; (2) partial-thickness wounds, where epidermis and deeper dermal layers are affected; (3) full-thickness wounds, where the subcutaneous fat and deeper tissue are ruptured [24].

### 2.2. Normal Wound-Healing Process

Wound healing is a complex, but a precisely synchronized, physiological process involving numerous factors such as multiple cells, mediators, growth factors, and components of the extracellular matrix (ECM). In general, the wound-healing cycle (Table 1) involves concurrent, but overlapping, stages of hemostasis, inflammation, proliferation, and remodeling [25].

In the hemostasis phase, a fibrin clot is formed at the site of injury to prevent blood loss and avoid microbial contamination by vasoconstriction [19]. Following this, the inflammatory phase begins almost immediately. In this process, the neutrophils engulf the existing bacterial cells in the wound and decontaminate the wound by secreting protease and antimicrobial peptides. The monocytes also differentiate into macrophages to kill apoptotic neutrophils, which secrete cytokines and several growth factors for an uninterrupted wound-healing process. This phase often takes 2–5 days to complete after skin damage [20]. The third phase, or the proliferative phase, typically occurs 5 days to 3 weeks after injury, and consists of cell proliferation and migration. During this phase, fibroblasts migrate to the wound site to produce ECM components (e.g., fibronectin, hyaluronic acid, collagen, and proteoglycan) and new blood vessels, as well as for re-epithelization [19]. Finally, in the remodeling phase, collagen III from the newly synthesized ECM is gradually replaced by collagen I, which has a more ordered lattice structure to impart the healed skin with enhanced tensile strength. Subsequently, this phase ranges from 3 weeks to 2 years (sometimes more than 2 years) after injury, and all the processes that were triggered after injury are terminated at this phase.

### 2.3. Wound Dressing

The main purpose behind wound dressing is to protect the wound from external contamination. It also retains hydration in the wound to enhance regeneration and prevent exposing the origin of the wound [26]. Therefore, wound-dressing materials should be biocompatible, semi-permeable to water and oxygen, hypoallergenic, and cost-effective. Hence, wound dressing requires technologically advanced dressing materials in contrast to traditional wound-dressing materials such as cotton and wool. The new materials are capable of not only preserving the wound environment but also of transferring active compounds to aid in the wound-healing process [14]. In this regard, various wound-dressing products, such as antibacterial creams, ointments, hydrogels, and antibacterial agents in combination with polymers, are currently available and consist mainly of biodegradable materials such as chitosan, hyaluronic acid, collagen, silicon, cellulose, and gelatin [27,28,29,30,31,32].

Quinolones [33], tetracyclines [34], cephalosporins [35], neomycin [36], and polymyxin B [36] are the most commonly used antibiotics in wound dressing because of their ability to prevent bacterial proliferation. However, repeated and inappropriate administration of antibiotics would increase the eruption of antibacterial resistance. Treatments using alternative antibiotics, non-antibiotic materials, and a combination of antibiotics with non-antibiotic materials such as essential oils, honey, and nanomaterials (e.g., Ag and Au), have been proposed for use in wound dressings to prevent such resistance [37].

### 2.4. Causes for Delayed Wound Healing

An acute wound typically takes 5 days to 2 weeks to enter the proliferative phase of the wound-healing cycle followed by a remodeling phase, which might extend for 2 years. However, if there is any abnormality in the physiological healing mechanism and it does not follow the usual healing pathway, then it might halt in one of the phases. This kind of situation in the wound-healing category named as chronic, non-healing wounds which normally failed to progress through a systematic process for normal wound healing. Chronic irritation, wound infection, persistence of bacterial proteins, and insufficient blood supply are major reasons for such delayed wound healing [19].

## 3. Nanomaterials in Wound Healing

The use of nanomaterials in wound healing is expanding rapidly; furthermore, nanotherapy-based wound-healing treatments are under clinical investigation [6,38,39]. The basic reason underlying the increased use of nanomaterials can be attributed to their physiochemical properties including nano size, large surface area, and high surface area-to-volume ratios. Moreover, the size and shape of nanomaterials are conducive to their use in wound healing because they play a role in active drug delivery, penetrability, and cellular responses [14]. Two types of nanomaterials are commonly used in wound therapy (Table 2): (1) nanomaterials with intrinsic properties that typically promote wound treatment, and (2) nanomaterials as vehicles for the delivery of therapeutic agents.

### 3.1. Intrinsic Antibacterial Agents in Wound Healing

#### 3.1.1. Metal and Metal Oxide Nanomaterials

The use of metal and metal oxide nanomaterials in wound-healing treatment is more advantageous than using traditional materials. Additionally, they possess similar qualities in wound-healing treatment and antibacterial action because of their intrinsic nature. Typically, the size, shape, surface functionalization, zeta potential, porosity, and hydrolytic stability of metallic nanoparticles affect the efficacy of nanomaterials in biological applications [16,17,18,19]. Among metallic nanoparticles, the most researched ones are silver (Ag) [83], gold (Au) [84], and zinc oxide (ZnO) [85] owing to their favorable physiochemical properties and antibacterial activity.

##### Silver Nanoparticles

Silver nanoparticles (Ag NPs) are well-known antibacterial agents against a wide range of bacterial infections and are commonly used to treat burns and wound infection. From ancient times (1850 BC), silver was applied to wounds in Egypt [86]. Even Hippocrates’ textbooks listed silver and its effect on wound-healing applications [86]. Over the past few years, the antibacterial property of Ag NPs has encouraged researchers to use them in various medical applications, including wound dressings, artificial implantation for preventing infection, and promoting wound healing [40,41]. Moreover, dressings based on Ag NPs do not result in any complications, even though they are employed in therapy for a prolonged period.

They have also been used in daily commodities, including textiles, clothing, air/water filtration, animal husbandry, food packaging, and medical applications [42,43]. In wound-healing therapy, Ag NPs-based material incorporated wound-dressing products such as Aquacel Ag^®^, DynaGinate™ AG Silver Calcium Alginate Dressing, CuraFoam™ AG Silver Foam Dressing, DynaFoam™ AG Bordered Silver Foam Dressing, Biatain^®^ Alginate Ag, and SilverIon^®^ are commercially available [41]. It is worthy to note that antibacterial mechanism of Ag NPs can be attributed to their nano size and increased surface area [41]. Because of these physiochemical properties, AgNPs can enter the microbe cell system by disrupting the cellular membrane and inhibiting the bacterial growth by causing intracellular damages.

Ag NPs and their conjugates with biopolymer (ABP) are also proved as non-cytotoxic and commonly accepted as Generally Recognized As Safe (GRAS) and synergistic biomaterials; their properties can also be used synergistically for wound dressing [41]. They promote wound healing in addition to preventing bacterial growth at the wound site. Therefore, ABP materials are regarded as significant to treatment application for both acute and chronic wounds. For instance, collagen, gelatin, silk, keratin, natural rubber latex, chitosan, starch, cellulose, and hyaluronic acid have all been used synergistically with Ag NPs in both in vitro and in vivo wound-healing applications [41]. Moreover, ABP-based biomaterials have been widely used in clinical trials and have shown promising outcomes. These ABP-based biomaterials are incorporated in commercially available wound-dressing products, including Promogran Prisma™ (collagen/Ag), ColActive^®^ Plus Ag (Porcine collagen/alginate ± Ag), and Biostep^®^ (Porcine collagen/alginate/CMC ± Ag) [44].

The incorporation of Ag NPs into wound dressing is one of the common techniques used for wound healing. The wound-dressing materials are poly (dopamine methacrylamide-*co*-methyl methacrylate) (MADO) with integrated Ag NPs that have been effective in inhibiting the growth of *E. coli*, *S. aureus*, and *P. aeruginosa* [45]. Moreover, the MADO-Ag NPs were also used for in vivo application of partial-thickness cutaneous wounds. Remarkably, the nanomaterials showed complete healing results and increased epithelization within 2 weeks compared with the results seen in the untreated group [45,46]. In another study, Zhou et al. [47] showed that silver–silver chloride nanoparticles coupled with reduced graphene oxide (Ag/AgCl/rGO) nanomaterials demonstrated enhanced wound-healing capability. In this case, Ag/AgCl/rGO nanomaterials produced more oxygen radicals or oxidative free radicals instead of releasing silver ions, and effectively showed antibacterial activity against both Gram-positive and -negative bacteria. Furthermore, the Ag/AgCl/rGO nanomaterials showed in vivo application in mice burn wounds with a faster rate of wound closure and enhanced re-epithelization.

##### Gold Nanoparticles

Another widely used nanomaterial in various biomedical applications such as wound healing, tissue regeneration, and targeted drug delivery constitutes gold nanoparticles (Au NPs) [84,87]. Their use in wound healing can be attributed to their chemical stability, their capacity to absorb NIR light, and their simple synthesis [14,88]. Additionally, the surface plasmon resonance property of Au NPs can be tuned with good effect for antibacterial and wound-healing properties [89]. Au NPs inhibited the growth of multidrug-resistant (MDR) pathogens, including *S. aureus* and *P. aeruginosa*, by binding to bacterial DNA and blocking the double-helix from uncoiling during replication or transcription, resulting in bacterial killing [14]. Another potential mechanism of Au NPs is that they can penetrate bacterial cells and modify bacterial membrane potential, which resulted in the inhibition of bacterial energy metabolism. It also inhibited the activity of ATP synthase enzyme, which leads to bacterial cell death [19]. Additionally, Au NPs prevent bacteria from forming reactive oxygen species and serve as antioxidants to help the wound-healing process [50].

Au NPs can easily be incorporated and cross-linked with collagen, gelatin, and chitosan for better wound-healing effects [51,52]. This process of functionalization helps the Au NPs to acquire properties such as biocompatibility and biodegradability. In this regard, Hsu et al. [53] showed that the wound-healing capability of Au NPs is substantially increased in combination with chitosan. Au NPs-chitosan increases the free-radical scavenging activity of Au NPs several fold with enhanced biocompatibility. Furthermore, in a rat surgical wound model, the nanocomposite increased epithelial tissue formation and improved hemostasis with a faster healing rate compared to chitosan alone. In a related study by Volkova et al. [54], it was shown that Au NPs combined with cryopreserved human fibroblasts (CrHFC-AuNP) applied topically to burn wounds exhibited an enhanced overall healing rate with reduced inflammatory phase and increased deposition of collagen. The study by Sherwani et al. [55] also demonstrated the ability of Au NPs to heal wounds.

##### Zinc Oxide Nanoparticles

Zinc oxide nanoparticles (ZnO NPs) are reliable inorganic antibacterial agents used in wound-healing applications. Zinc is a long-lived element in living cells that is important for wound healing, particularly in delayed wound healing and burns [19]. The antibacterial activity of ZnO NPs depends on their ability to disrupt the bacterial cell membrane [85,90]. The combination of ZnO NPs with a chitosan hydrogel demonstrates optimal antibacterial activity with low toxicity and renders the composite an ideal material for wound dressing [63]. In this respect, Balaure et al. [81] reported that the wound dressing consisting of collagen with ZnO NPs and 1% orange essential oil demonstrates accelerated wound closure and also inhibits bacterial growth with excellent biocompatibility both in vitro and in vivo. Likewise, the work by Gao et al. [82] confirmed the wound-healing ability of ZnO NPs as an antimicrobial tissue adhesive with an in vivo study using a skin wound mouse model. However, the intrinsic toxicity of ZnO NPs remains to be a drawback, which hinders their application in wound healing and requires further investigation.

Significant research interests are also being developed for other metal and metal oxides including copper, titanium, magnesium oxide (MgO), iron oxide (Fe_2_O_3_), aluminum oxide (Al_2_O_3_), and copper oxide (CuO) in wound-healing materials [5].

#### 3.1.2. Non-Metallic Nanomaterials

Non-metallic nanomaterials were proposed as curing agents for wound healing. Among them, carbon-based nanomaterials, including fullerenes, carbon nanotubes, and graphene, have demonstrated tremendous potential for use as nanomedicine in various biomedical applications such as bioimaging, tissue regeneration, and controllable medicinal products [67]. Fullerenes, based on their antioxidant and anti-inflammatory properties, showed promising results for wound-healing applications [67]. Because of their antioxidant properties, fullerenes are capable of scavenging and detoxifying reactive oxygen species (ROS) and reactive nitrogen species, which are advantageous for wound-healing applications. However, when associated with cells, fullerenes may be aggregated in the cell system. In addition, their aggregation in the cell system can be prevented by functionalizing with hexa-dicarboxyl, tris-dicarboxyl, and γ-cyclodextrin [69]. Additionally, fullerene, multiwalled carbon nanotubes, and graphene oxide (GO) also displayed remarkable wound-healing properties [13]. A recent study by Khan et al. [70] successfully reported the use of GO nanosheets for photothermal treatment of bacterial and fungal wound infection. The synergistic effect of the nanomaterials healed the wound comparatively faster than their individual application.

Polymeric nanoparticles can be used as wound dressings or as delivery vectors owing to their antibacterial and pro-wound-healing properties [91]. Among these nanoparticles, naturally occurring polymers such as chitosan were mainly studied for wound-healing applications. Chitosan was once the favored source for wound-healing applications because of its biocompatibility, biodegradability, and antibacterial properties [27]. In addition, chitosan-associated nanocomposites are generally more effective than chitosan itself for wound-healing applications. Hajji et al. [61] reported that the nanocomposite with chitosan-polyvinyl alcohol-Ag NPs exhibited superior antioxidant and antimicrobial properties than the simple chitosan polymer. Additionally, it significantly enhanced the in vivo wound closure with reduced cytotoxicity. Furthermore, Holban et al. reported that the polylactic acid-chitosan-magnetite-eugenol nanospheres demonstrated comparatively better inhibition of biofilm formation than chitosan itself, while showing endothelial proliferation [62]. Several researchers have studied cellulose for its antibacterial and wound-healing properties. In this respect, Singla et al. [92] showed the in vivo diabetic wound-healing potential of nanobiocomposites comprising nanocrystals from bamboo cellulose and Ag NPs.

Black phosphorus (BP) has also emerged recently as an excellent nanomaterial for various biomedical applications including wound healing. Huang et al. [59] recently reported that water-based BP nanosheets could be used as a moldable platform for wound-healing applications. These BP-based materials showed excellent biocompatibility with NIR-mediated photothermal effects that efficiently prevented bacterial infections and subsequently promoted wound healing; this signifies enormous clinical potential in wound-care management. In addition, Mao et al. [60] reported that BP embedded hydrogel possesses excellent potential for wound healing and sterilization. Singlet oxygen (^1^O_2_) generation ability of BP-based nanomaterial under simulated visible light rapidly caused them to kill bacteria. This hydrogel also has high repeatability in terms of antibacterial activity and biocompatibility without causing considerable defects or damage to major organs such as the heart, liver, spleen, lung, and kidney in rats during the wound-healing process. Therefore, the BP-based nanocomposite has potential applications in photothermal-mediated antibacterial activity and wound healing.

### 3.2. Nanomaterials as Nanocarriers for Wound Healing

Nanomaterials may also serve as nanocarriers for therapeutic agents rather than acting as intrinsic antibacterial agents and aid in wound-healing processes. The major drawback of conventional antibiotic therapy in non-healing chronic wounds of biofilm-related infections is the insufficient delivery of antibiotics to target cells. The explanation for this can be attributed to the complex matrix of biofilm-forming extracellular polymeric substance (EPS) [5]. This phenomenon decreases the therapeutic impact of available medications and also enhances drug resistance. Therefore, major efforts were made toward the controlled and sustained release of drugs to the target site. Nanomaterials in this regard may be the future nanocarriers for the targeted delivery of therapeutic agents. In addition, the delivery of the nanomaterial-mediated therapeutic agents increases the antibacterial activity relative to the antibiotic itself. The various explanations behind this increase in antibacterial activity include the release of drugs in a controlled manner with optimal concentration, shielding of drugs from enzymatic inactivation, and delivery of drugs to specific target sites [59].

#### 3.2.1. Nanomaterials Combined with Antibiotics

The size of the nanomaterials aids in their easy penetration of the biofilm-forming bacterial cell membrane or EPS. Several nanomaterials, such as liposomes, polymeric nanoparticles, and peptide nanostructures, with biocompatible and biodegradable properties have been effectively used as nanocarriers for antibiotics to reach the desired target site for wound-healing applications [60,75,76,77,93,94]. The phospholipid bilayer structure of liposome nanoparticles enables their use in numerous biomedical applications as a drug-delivery carrier. For example, the β-lactam antibiotic and piperacillin encapsulated within liposomes could be protected from hydrolysis by Staphylococcal β-lactamase [60]. This contributed to higher antibacterial activity against *S. aureus* for piperacillin encapsulated within liposomes relative to the piperacillin alone. In a related study, Mugabe et al. [75] showed that gentamicin antibiotic encapsulated within liposomes resulted in considerably higher antimicrobial activity against *P. aeruginosa* biofilms than gentamycin alone. The daptomycin-encapsulated liposomes also showed encouraging inhibition of biofilm growth from *S. aureus* at the infection site in a mouse model of subcutaneous infection [76]. Nonetheless, given its promising findings, further research is required because antibiotic-encapsulated liposomes at present demonstrate relatively low chemical and physical stability.

Poly(lactic-*co*-glycolic acid) (PLGA), among polymeric nanoparticles, has been widely studied for wound-healing applications [77]. Like the liposome, its biodegradability and biocompatibility property make it an ideal nanomaterial for wound-healing applications. There are numerous reports [5,77] regarding the antibacterial activity of antibiotics encapsulated-PLGA against *E. coli*, *S. aureus*, and *P. aeruginosa*. In addition to polymer and liposome nanomaterials, a new class of nanocarrier and lipid-polymer hybrid nanoparticles has recently emerged as an alternative to polymer and liposome nanomaterials [93,94]. Such nanoparticles combine the advantages of both liposomes and polymeric nanoparticles. In addition, functionalized antibiotics with nanoparticles may also facilitate wound-healing applications. In this regard, the study by Chen et al. [95] showed that functionalized gold nanodots with antimicrobial peptides would inhibit the growth of drug-resistant bacteria and promote wound healing in a rodent wound model. In another study, Au NPs combined with epigallocatechine gallate and α-lipoic acid accelerated healing in diabetic ulcer wounds [56]. In this respect, a market product, NanoRepair Q10^®^ from the company Dr. Rimpler [96], is available in the market where the formulation of solid-lipid nanoparticles with Q10 was used for topical therapeutic or cosmetic purposes.

Moreover, the purpose of using nanomaterials regarding galenic formulations for topical administration is to protect active pharmaceutical ingredients (APIs) from degradation and targeted delivery for local action [97]. Additionally, this strategy also helps to prevent APIs from penetrating into the bloodstream, which in turn reduces the risk of unintended toxicity. The advantages of using nanomaterial for galenic formulations for topical administration are that they are generally biocompatible, biodegradable, and capable of loading both hydrophilic and lipophilic APIs. In this respect, liposomes, solid-lipid nanoparticles, polymeric nanoparticles have already been utilized for clinical formulation [20]. Different indication issues will be selected regarding the use of nanomaterials for galenic formulations in wound-healing applications such as photoaging reduction, dermal fungal infections treatment, burn wound healing, and others.

This revolutionary approach to loading and delivering antibiotics using nanoparticles to the target site has great potential, as this can achieve various goals such as loading efficiency, desirable dose, appropriate stability, low water solubility, less harm to healthy tissues, drug release property, and prevent rapid degradation in the cell system. Nonetheless, despite its potential, only a few clinical trials have been conducted in this regard because of its high cost and inefficient drug loading [98]. Therefore, factors [86,99] such as time-released delivery of drugs by nanoformulation, optimization of surface characteristics, healing time, cost-effectiveness, scarring reduction, international evaluation methods for measuring their toxicology, and large-scale industrial production should always be considered for effective clinical application of nanomaterial combined with antibiotics. Therefore, further research is required regarding higher antibiotic encapsulation efficiency and prevention of premature drug release.

#### 3.2.2. Nanomaterials Containing Nitric Oxide

Nitric oxide (NO) is an inherent pro-wound–healing agent, since it exerts a wide range of antimicrobial activity by interfering in the DNA replication and respiration processes [100]. This is also effective in diffusing biofilm formation, which in turn aids in the wound-healing process [101]. Nevertheless, its short half-life along with its instability in the tissue in vivo hampered its therapeutic wound-healing applications. Therefore, nanomaterials as a nanocarrier have tremendous potential for the controlled release of NO with regard to the antimicrobial treatment of wound infections.

Nevertheless, the nanomaterial carrier for the NO delivery system should have the following properties of high loading capacity, with extended release time, and low cytotoxicity [78,102]. In this regard, Nurhasni et al. [78] reported nanoparticles of NO-releasing PLGA—polyethylenimine (PEI) to assess wound-healing efficacy in wounds infected with methicillin-resistant *S. aureus* ( MRSA). The nanoparticle system of PLGA-PEI allowed prolonged NO, which facilitates antibacterial activity and wound healing. In another study, NO-releasing silica nanoparticles showed effective antibacterial activity by destroying bacterial cells within established biofilms [80]. Additional in vivo experiments with NO-releasing nanoparticles made of silane hydrogels using murine wound models demonstrated their efficacy against wound infections [103,104]. Therefore, the potential NO-releasing nanoparticles may be a reliable alternative to antibiotics for treating wound infection. However, the main challenge with regard to the NO-based therapy is to maintain an optimal concentration of NO in the target wound site because fluctuating levels (either too high or too low) of NO may hinder the wound-healing process.

## 4. Nanomaterial-Based Scaffolds for Wound Healing

In another application of wound healing, nanomaterials are successfully used to generate nanopolymeric scaffolds that mimic properties such as the fibrous nature and nanoscale features of ECM [6]. Electrospinning, self-assembly, and separation of phases are among the different techniques commonly used to create nanomaterial-based scaffolds. Of these processes, electrospinning for nanoscaffold manufacturing is the most commonly used technique employed by researchers [105,106]. This technique succeeds in producing porous polymeric nanofibers, which can produce fibroblasts in wounds and exhibit similar physical and structural properties to those of ECM. In this regard, Shahverdi et al. [79] made successful use of PLGA/silk fibroin as hybrid scaffolds by developing fibroblast cells to enhance the healing of diabetic wounds. In another study, chitosan-poly-vinyl alcohol nanofibrous blend scaffolds with electrospun were successfully implemented to treat diabetic wounds in rats for higher healing levels compared to controls [64]. Using in vivo analysis in Wistar rats, Dong et al. [48] showed the efficacy of electrospun nanofiber membrane with Ag NPs with reduced inflammation and increased wound healing, coupled with low cytotoxicity and long-term antibacterial action. Furthermore, the work by Fu et al. [107] showed accelerated dermal wound healing by using electrospun curcumin-loaded poly(ε-caprolactone)-poly(ethylene glycol)-poly(ε-caprolactone) fibrous mats. This electrospun composite scaffold showed low cytotoxicity and anti-oxidant effect in vitro, and enhanced wound healing in vivo. Certain polymers with nanodimension, such as dendrimers with anti-inflammatory properties can also be incorporated into wound dressings. For example, electrospinning technique generated gelatin–dendrimer nanofibers treated with polyethylene glycol (PEG) for semi-interpenetrating networks (sIPNs) showed excellent wound-healing property [108]. Moreover, Ag NPs were incorporated within the sIPNs network for adding antibacterial activity.

Many other studies concerning the use of nanoparticle-containing scaffold for wound-healing applications are available [13]. In this regard, the wound-healing potential of nanocomposites comprising chitosan, silver, copper, and zinc nanoparticles was assessed in adult rats. In a related study, nanocomposites based on chitosan and copper nanoparticles demonstrated their capacity in the proliferative phase of the wound-healing process [65]. Surface-modified magnetite (Fe_3_O_4_@C16) nanoparticles functionalized with eugenol and limonene showed antibacterial and anti-adherence properties, which are essential for wound regeneration applications [71]. Similarly, gentamicin-loaded ZnO NPs with chitosan in three-component gel [66] showed synergistic antibacterial activity with superior growth-inhibition ability for *S. aureus* and *P. aeruginosa* compared to the gentamicin control; therefore, this result indicated that such a nanocomposite is beneficial for wound-healing applications involved in the infection of pathogens at the wound site. In another study, Ag NPs were distributed in a polyethylene cloth and in both in vitro and in vivo wound models [109]. Toxicity and cells deaths were not observed in the healed skin samples. Moreover, the inclusion of Ag NPs allowed fast regeneration of cutaneous layer in vivo. A similar kind of study was attempted by Auddy et al. [110], where a new cationic biopolymer guar gum alkylamine with Ag NPs for enhanced wound closure. Using the same combined strategy, a preclinical study of fibrous mats/scaffolds for wound dressing material was evaluated using electrospun consisting of PVA (polyvinyl alcohol) and chitosan oligosaccharides with Ag NPs (15–22 nm) [111]. The material showed excellent antibacterial activity against *E. coli* and *S. aureus* within the wound site without showing toxicity. The nanotopography of a wound-dressing scaffold is one of the prime objectives of researchers currently to maintain a high rate of wound closure coupled with reduced scar formation. In this regard, Kim et al. [112] demonstrated that the orientation and density of nanogrooves are vital for fibroblast migration during wound healing, and thus highlighted the relevance of the nanotopography of the scaffold in wound healing.

## 5. Nanomaterial-Based Growth Factors for Wound Healing

Growth factors can be defined as biologically active polypeptides which play a pivotal role in modulating and coordinating cellular processes such as cell growth, differentiation, and migration during all stages of wound healing [6]. It is worthy to note that recombinant human platelet derived growth factor (rhPDGF) is the only available growth factor approved by the FDA. Although other growth factors such as recombinant human epidermal growth factor (rhEGF) and recombinant human vascular endothelial growth factor (rhVEGF) are available [6], they are still lacking the FDA approvals due to lack of large clinical evidence.

Another major hurdle regarding growth factors is that they fail to protect themselves from enzymatic degradation in the proteolytic wound environment [6]. To resolve this issue, PLGA NPs, chitosan NPs, and solid-lipid NPs could be used to protect the growth factors from enzymatic degradation (Table 3). Chu et al. [113] successfully showed that PLGA NPs could be used as a carrier in the preparation of rhEGF nanoparticles. In a similar way of study, rhEGF growth factor was incorporated into chitosan NPs within a fibrin gel for stable and sustained release of the growth factor [114]. Furthermore, Xie et al. [115] successfully employed a hybrid composite of PLGA nanoparticles embedded in chitosan–poly(ethylene oxide) nanofibers for dual growth factor release of VEGF and PDGF. Despite the effectiveness of growth factors in the wound-healing process, multiple studies are still required to solve their proteolytic enzymatic degradation with the help of nanomaterials before they can be used clinically.

## 6. Nanomaterial-Based Innovative Strategies

### 6.1. Nanomaterials for Antibacterial Hyperthermia Treatment

One of the groundbreaking nanomaterial-based approaches developed in the last few years is the treatment of hyperthermia to cure or prevent bacterial infections. The basic concept is to damage the bacterial cells by inducing irreversible thermal energy with an externally applied energy source such as NIR light or high frequency alternating magnetic field (AMF) [5]. Initially, the nanomaterials absorb the external energy and generate heat, which in turn increases their surface temperature and eventually kills the bacteria. It noteworthy that the bacteria seemed to have died at above 55 °C due to the denaturation of heat-shock proteins [116].

**Figure 1:**
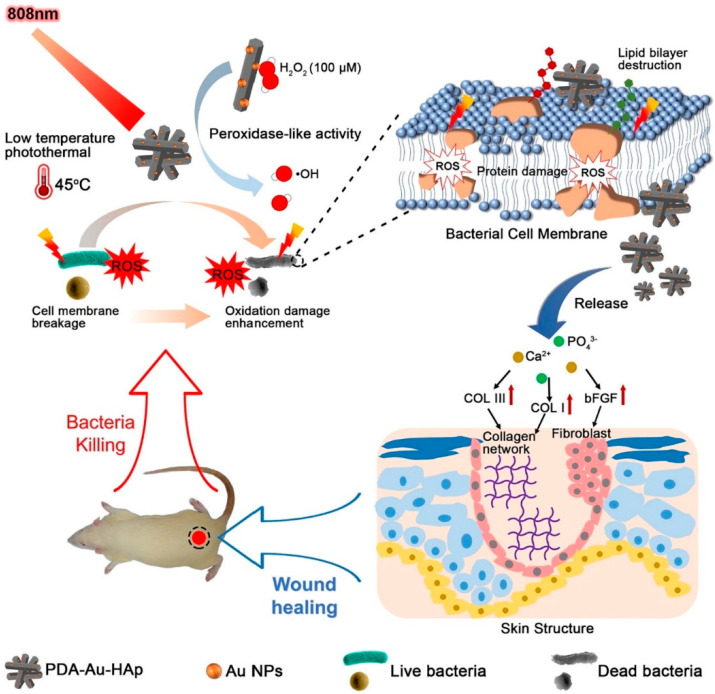
Schematic illustration of photothermal therapy-mediated antibacterial effects and promotion of wound healing in vivo. Reproduced with permission from Ref. [57], copyright 2018, Elsevier.

The photothermal effect exhibits high light–thermal conversion efficiency under the irradiation of NIR light [116]. Additionally, the NIR light within its range of wavelength (700–1400 nm) can penetrate mammalian cells, causing minimal damage to normal cells. Therefore, photothermal therapy (PTT) is now regarded as a safe, efficient, and innovative strategy to deal with bacterial infections or wound healing (Figure 1). Among the suitable nanomaterials, Au, iron oxide, graphene, carbon nanotubes (CNTs), and BP have been mostly used as photothermal agents for NIR light-mediated photothermal activity due to their optical property. One of the good examples of the use of nanomaterials for antibacterial photothermal treatment is described in the study by Xu et al. [57]. The polydopamine-assisted hydroxyapatite (PDA@Au–Hap) nanocomposite not only enhances the photothermal antibacterial activity at 45 °C, but also avoids unnecessary damage to normal tissues. Moreover, it also aids the formation of granulation tissue with collagen-synthesis and wound-healing applications. Another study by Zhao et al. [117] described the treatment of an in vivo diabetic wound infection model with BSA–CuS-based photothermal therapy. The nanomaterial demonstrated a faster healing rate than that seen in the control group. Moreover, Chiang et al. [118] successfully developed a hybrid microsphere with a shell of PLGA and aqueous cores of polypyrrole nanoparticles and vancomycin. Here, the polypyrrole nanoparticles were regarded as a photothermal agent. The polypyrrole-based combination synergistically eradicated bacteria in the wound abscesses of mice, which is greater than the sum of two individual treatment strategies.

Another interesting nanomaterial-based system is AMF-based magnetic hyperthermia. In this system, the heating potential of magnetic nanoparticles (MNPs) was used with the aid of high-frequency AMF (>100 kHz) [119]. The MNPs absorb the electromagnetic radiation generated by the AMF, and subsequently transmit highly localized heat onto the nanomaterial surface, which has the potential to kill a variety of bacterial pathogens. In this regard, a recent combined approach of magnetic hyperthermia with d-amino acids was found to successfully inhibit and disperse biofilms [72]. Initially, the D-amino acids were used to disrupt the EPS of biofilm, followed by AMF exposure. This combinational approach completely eradicated *S. aureus* biofilms with no toxicity to mammalian cells in vitro. This approach was further demonstrated by Thomas et al. [73], where the AMF-based hyperthermia was successfully applied to destroy an in vitro culture model of *S. aureus*. Furthermore, Kim et al. [74] successfully applied this AMF-based magnetic hyperthermia to disrupt biofilms produced by *S. aureus* in studies conducted both in vitro and in vivo study. However, precautions need to be taken, as overdoing can damage normal tissue cells. Therefore, critical design is always necessary for its safe application.

### 6.2. Nanomaterial-Based Gene Nanotherapy

Gene-activated matrix therapy has evolved recently for the regeneration of skin, bone, and cartilage [120]. The stability of DNA is the main advantage of gene therapy in wound-healing applications. However, current techniques (e.g., direct injection, transfer by gene gun, and electroporation) used to deliver DNA to wound sites need repeated injections or are hindered by inconsistent and short-term gene expression [121]. This can be resolved with nanomaterials such as electrospun nanofibrous meshes that can be used as matrices for gene encapsulation and wound-dressing material. In this regard, Kobsa et al. [122] showed that electrospun scaffolds of a blend of poly(lactic acid) (PLA) and poly-ε-caprolactone (PCL) can be successfully used for the localized delivery of a DNA plasmid encoding keratinocyte growth factor; this also has the potential to treat cutaneous wounds. Moreover, spherical nucleic acid gold nanoparticle conjugates were effectively used for efficient siRNA delivery in vivo [58]. Another study by Tong et al. [49] showed that single-stranded DNA (ssDNA)-guided Ag NPs on GO (ssDNA-AgNPs@GO) showed excellent antibacterial activity along with their ability to cure the wound infection caused by *S. aureus* bacteria. However, more efficient, gene-based technologies for wound healing are still required.

## 7. Conclusions and Future Perspectives

The main purpose of this review was to highlight the advantages of using nanomaterials for the wound-healing process. It is noteworthy that the unique physiochemical properties of nanomaterials render them ideal candidates for wound-healing applications. The nanomaterial-based wound-healing process also proved to be more effective than conventional wound therapy, which is primarily based on dressing. Nanomaterials can alter one or more wound-healing phases of the wound-healing process, since they possess antibacterial, anti-inflammatory, and anti-proliferative properties. However, some key issues remain to be resolved for the successful application of nanomaterials in clinical wound-healing therapy. The toxicity of nanomaterials is one of the principal concerns, because it can have possible adverse effects on human cells. Therefore, this needs to be resolved at the outset for more advanced wound-healing tests or clinical trials. Another problem is that with the in vivo model, there is less knowledge about non-material mediated wound-healing applications. Most of the reported studies of nanomaterial-based wound-healing applications are based on in vitro studies or mostly rely on single-target bacteria. Therefore, more in-depth studies employing both Gram-positive and -negative bacterial strains or skin microbiota are required for in vivo wound-healing applications. Future efforts should focus at enhancing site specificity and targeting efficiency for more effective wound-healing applications as per the nanomaterial used. Therefore, researchers should aim at developing biocompatible and biodegradable nanomaterials capable of correcting all phases of wound healing.

## Figures and Tables

**Table 1:** Nanomaterials that showed favorable effects in different wound-healing phases.

Phases	1st: Hemostasis (Few Minutes)	2nd: Inflammatory (0–5 Days)	3rd: Proliferative (5 Days–3 Weeks)	4th: Remodeling (3 Weeks–≥2 Years)
Specific events	- Vasoconstriction - Platelet aggregation - Blood clotting	- Recruitment of neutrophils and macrophages - Phagocytosis - Secretion of various growth factors and cytokines	- Fibroblast proliferation - Collagen synthesis - Angiogenesis - Re-epithelization	- Replacement of collagen III by collagen I - ECM remodeling - Scar formation
Nanomaterials	- Polymeric NPs - ZnO NPs - CeO_2_ NPs	- Polymeric NPs - Au NPs - Ag NPs - Dendrimers - Liposomes - Fullerenes - Polymeric nanoscaffolds	- Polymeric NPs - Dendrimers - Iron oxide NPs - Au NPs - Liposomes - Cu NPs - Ag NPs - Ceramic NPs	- Polymeric NPs - Iron oxide NPs - Polymeric nanoscaffolds

NPs, nanoparticles; ECM, extracellular matrix.

**Table 2:** Various nanomaterials and their role in wound healing.

Nanomaterials	Role in Wound Healing	References
Ag NPs	Intrinsic antibacterial agent Conjugated with polymer in scaffold for synergistic antibacterial activity Synergistic activity in nanocomposite for gene nanotherapy	[40,41,42,43,44,45,46,47] [48] [49]
Au NPs	Intrinsic antibacterial agent Nanocarriers for antibiotics to reach target site Synergistic activity in nanocomposite for hyperthermia treatment Effectively used siRNA delivery for gene nanotherapy	[50,51,52,53,54,55] [56] [57] [58]
BP	Mostly used as photothermal agent for hyperthermia treatment Embedded in hydrogel or as a moldable platform for wound healing	[59] [60]
Chitosan	As wound-dressing material Used with metal, metal oxide for synergistic antibacterial and wound-healing properties Conjugated with other nanomaterials in scaffold formation and antibacterial activity	[27,61,62] [53,61,63] [64,65,66]
CNTs	Intrinsic antibacterial agent Photothermal agent for hyperthermia treatment	[13,67] [68]
Fullerene	Intrinsic antibacterial agent	[67,69]
Graphene	Conjugated with metal, metal oxide for synergistic antibacterial and wound-healing properties Photothermal agent for hyperthermia treatment Synergistic activity in nanocomposite for gene nanotherapy	[47] [70] [49]
Iron oxide NPs	Synergistic antibacterial activity in scaffold AMF-mediated hyperthermia treatment	[71] [72,73,74]
Liposomes	Primarily used as nanocarriers for antibiotics to reach target site	[60,75,76]
PLGA NPs	Nanocarriers for antibiotics to reach target site Nanocarriers for NO release at target site Hybrid scaffold material	[77] [78] [79]
Silica NPs	Nanocarriers for NO release at target site	[80]
ZnO NPs	Intrinsic antibacterial agent for wound dressing Conjugated with polymer in scaffold for synergic antibacterial activity	[81,82] [66]

BP, black phosphorus; CNTs, Carbon nanotubes; NO, Nitric oxide; NPs, Nanoparticles; PLGA, Poly(lactic-*co*-glycolic acid).

**Table 3:** Nanomaterial-based scaffolds for wound healing.

Scaffold Material	Activity	References
PLGA/silk fibroin	Diabetic wound healing	[79]
Chitosan-PVA	Treated diabetic wounds in rats	[64]
Nanofiber with Ag NPs	Reduced inflammation, increased wound healing, low cytotoxicity and long-term antibacterial action	[48]
Fe_3_O_4_@C16	Showed antibacterial and anti-adherence properties, which are essential for wound regeneration	[71]
Gel containing gentamicin, ZnO NPs and chitosan	Showed synergistic antibacterial activity and can be beneficial for wound healing	[66]
Ag NPs in polyethylene cloth	Fast regeneration of cutaneous layer in vivo	[109]
Guar gum with Ag NPs	Enhanced wound closure	[110]
PVA, chitosan oligosaccharides with Ag NPs	Excellent antibacterial activity within wound site and no toxicity	[111]

Fe_3_O_4_@C16, Surface modified magnetite; NPs, Nanoparticles; PVA, Polyvinyl alcohol; PLGA, Poly(lactic-*co*-glycolic acid).
